# Cardiorenal metabolic biomarkers link early life stress to risk of non-communicable diseases and adverse mental health outcomes

**DOI:** 10.1038/s41598-020-69866-3

**Published:** 2020-08-06

**Authors:** Janet Poplawski, Ana Radmilovic, Tony D. Montina, Gerlinde A. S. Metz

**Affiliations:** 1 Canadian Centre for Behavioural Neuroscience, Department of Neuroscience, University of Lethbridge, 4401 University Drive, Lethbridge, AB T1K 3M4 Canada; 2 Department of Chemistry and Biochemistry, University of Lethbridge, 4401 University Drive, Lethbridge, AB T1K 3M4 Canada

**Keywords:** Experimental models of disease, Outcomes research, Translational research, Metabolomics

## Abstract

Stress is one of the most critical determinants of lifetime health and increases the risk of chronic non-communicable diseases. To gain insight into underlying environment-gene interactions, we analyzed the cardiorenal metabolome of adult mice exposed to multidimensional early-life transportation stress. Using proton nuclear magnetic resonance (^1^H NMR) spectroscopy, we show that early life stress permanently programs metabolic pathways in somatic organs linked to cardiorenal and mental health disorders in later life. Heart and kidneys of stressed mice revealed robust metabolic markers linked to abnormal energy metabolism, branched-chain amino acid biosynthesis and degradation, methylhistidine metabolism, arginine and proline metabolism, glycine and serine metabolism, and aminoacyl-tRNA biosynthesis. These markers were strongly associated with anxiety-like behaviours. Dysregulation of energy and protein metabolism suggests an increased risk of metabolic diseases like insulin resistance, cardiorenal syndrome, diabetes, and obesity. These findings provide novel insights into the direct effects of early life stress on cardiorenal metabolism and are consistent with prior observations of increased non-communicable disease risk in stressed populations. Thus, stress-associated metabolic signatures in somatic organs may provide early predictors of health risks in later life and reveal new candidates for peripheral biomarker detection with diagnostic value.

## Introduction

Chronic non-communicable diseases (NCDs) are a major public health problem with a high economic cost to health systems. At least a third of all individuals with one NCD are subsequently diagnosed with additional chronic non-communicable conditions^1^. Cardiovascular diseases (CVDs) are the most prevalent category of NCDs, accounting for nearly half of all NCD-related mortalities^2^. CVDs often co-occur with mental health disorders^3,4^ and impaired renal function, with CVDs being twice as common in patients with chronic kidney disease and advancing at twice the rate^5,6^. Moreover, individuals suffering from emotional disorders are twice as likely to experience myocardial infarction relative to the general population, and this cardiovascular comorbidity significantly increases mortality in patients with depression^4,7^. An estimated 40% of individuals with chronic CVDs also suffer from anxiety, and overall anxiety levels are 60% higher in CVD patients relative to healthy adults^8,9^. The comorbidity of adverse cardiovascular events, renal dysfunction, and affective disorders suggests shared pathways in the pathogenesis of these conditions.

An individual’s susceptibility to NCDs is predominantly, although not exclusively, environmentally determined^9,10^. However, traditional risk factors for chronic diseases such as poor nutrition, inactivity, and adiposity do not fully account for the excess burden of NCDs in the population. Thus, other factors such as stress may be precipitating events in the development and progression of chronic non-communicable conditions. Growing evidence implicates stress and its effector system, the hypothalamic–pituitary–adrenal (HPA) axis, in the pathophysiology of cardiorenal and affective disorders^11–15^. Repeated exposure to prenatal stress has been shown to program cardiovascular sensitivity to subsequent stress; specifically, prenatally stressed animals display increased blood pressure and blood pressure variability in response to subsequent acute stressors, as well as tachycardia and hypertension during the post-stress period^16^. While prenatal and early postnatal stress each can dampen HPA axis responses in the offspring^17^, synergistic effects of both may potentially support stress coping mechanisms^17^. Moreover, circadian disorganization has been associated with an increased risk of cardiorenal diseases, metabolic syndrome, and anxiety-like behaviours in both human^18^ and animal studies^19,20^, mediated by altered rhythms of gene expression and endocrine factors^21^. Notably, distinct organ systems exhibit transient critical periods of heightened plasticity and increased vulnerability to environmental insults during infancy^22^. Accordingly, early life stress can also have lasting deleterious effects on physiology and cardiovascular^10^ and renal diseases^23,24^, metabolic disease^25^, behaviour and risk of affective disorders^26^. The mechanisms underlying the association with cardiorenal and emotional disorders, however, are still poorly understood.

Comprehensive metabolic profiling of biological samples has emerged as a promising approach to characterizing the pathophysiological consequences of maladaptive gene-environment interactions. Metabolic phenotypes represent an organism’s downstream physiological responses to epigenetically regulated cellular processes; therefore, metabolic profiling is a powerful tool for identifying stress-induced adverse health outcomes. Here, we used proton nuclear magnetic resonance (^1^H NMR) spectroscopy to determine whether early life stress in a mouse model permanently alters cardiorenal metabolism. We identified clearly distinguishable cardiorenal metabolic signatures and mechanistic pathways linking impaired cardiorenal function to mental health disorders.

## Materials and methods

### Experimental design and multidimensional early life stress

Twenty-three male C57BL/6 mice (*Mus musculus*) were used. A multidimensional shipment stressor was chosen as a clinically valid stress model because it occurs prominently in human populations^27,28^ and in laboratory animal and livestock transportation^29–31^. Stressed animals (n = 14) were shipped from Charles River Laboratories (Charles River Laboratories, QC, Canada) via 3.25 h of ground transportation and a 5-h flight as airfreight. These pups and their mothers were shipped from 7:30 am to 7:30 pm on postnatal day (P)12. Control animals (n = 9) were bred and raised under controlled conditions at the local vivarium but their ancestors were purchased from Charles River Laboratories. All pups remained with their mother until weaning at 9.6 g, after which animals were housed in groups of at least two.

Anxiety-like behaviour was assessed at 10:00 am on P25 using a standard open field task. Video recordings were made to determine the number of central squares entered in a 1-min session. To assess the impact of multidimensional early-life transportation stress on cardiorenal metabolism, kidney and heart tissues from adolescent (P50) mice were extracted, weighed, and processed for metabolomic profiling by ^1^H NMR spectroscopy. All procedures were approved by the University of Lethbridge Animal Care Committee and all methods were in compliance with the guidelines by the Canadian Council on Animal Care.

### Sample collection and preparation

Mice received an intraperitoneal overdose of sodium pentobarbital (150 mg/kg; Euthansol; Merck, QC, Canada). Kidney and heart tissues were extracted, weighed, and stored at – 80 °C. To isolate water-soluble metabolites for NMR analysis, tissues were thawed at room temperature and subjected to methanol-based protein precipitation as well as chloroform-based lipid extraction. Finally, samples were centrifuged at 12,000 g for 5 min at 4 °C to precipitate any particulate matter, and 550 μL of the supernatant was transferred to a 5-mm NMR tube for NMR analysis.

### NMR data acquisition and processing

Data were acquired using a 700 MHz Bruker Avance III HD spectrometer (Bruker, ON, Canada). Spectra were obtained using a Bruker triple resonance TBO-Z probe with the outer coil tuned to the nuclei of ^1^H, ^31^P and ^2^H and the inner coil tuned to the ^13^C nucleus. The standard Bruker 1-D NOESY gradient water suppression pulse sequence ‘noesygppr1d’ was used with a mixing time of 10 ms. Each sample was acquired with 128 K data points, a sweep width of 20.5136 ppm, and a recycle delay of 4 s. Tissue samples were run for 512 scans to a total acquisition size of 128 k. The resulting spectra were then zero filled to 256 k, line-broadened by 0.3 Hz, transformed to the frequency domain, phased, and baseline-corrected. Spectral processing was performed using the Bruker Topspin software (version 3.2, patch level 6), after which spectra were exported to MATLAB (MathWorks, MA, USA) for spectral binning, data normalization, and scaling. Spectra were binned using Dynamic Adaptive Binning^32^. Datasets were normalized using the Constant Sum method^33^ to remove effects of imperfect water signal suppression. The dataset was then Pareto-scaled (mean-centered and divided by the square root of each variable’s standard deviation). All peaks were referenced to TSP (0.00δ).

### Statistical analyses

Spectral bins were first analyzed for all comparison groups and classified as either significant or non-significant using a decision tree algorithm and a Mann–Whitney U (MW) test. One hundred and seventy bins were initially included in the kidney analyses and 347 spectral bins were included in the heart analyses. All *p*-values obtained from these analyses were Bonferroni-Holm corrected for multiple comparisons. Variation in spectral data was visualized using Principal Component Analysis (PCA), Partial Least Squares Discriminant Analysis (PLS-DA), and Orthogonal Projection of Latent Structures Discriminant Analysis (OPLS-DA) using Metaboanalyst. Double cross-validation and permutation testing (2,000 iterations) were performed to validate statistically significant PLS-DA and OPLS-DA results. Variable importance in the projection analysis was performed and plots were made using the weighted sum of squares of the PLS loadings.

Metabolite set enrichment analysis and pathway topology analysis were performed using Metaboanalyst. Metabolic pathway analysis identified the most relevant pathways based on the Kyoto Encyclopedia of Genes and Genomes (KEGG) pathway database (*Mus musculus*) and the Over Representation Analysis^33^ selected, using a hypergeometric test. The number of central squares entered by each animal in the open field apparatus was related to metabolic changes using Pearson *R* correlations.

### Metabolite identification

The Chenomx 8.2 NMR Suite (Chenomx Inc., AB, Canada) was used to identify metabolites present in tissue spectra. Metabolite identities were validated using the Human Metabolome Database.

## Results

### Stress altered the tissue phenotype

Early life stress led to abnormal kidney and heart weights. Specifically, absolute kidney weights were significantly increased [*t*(21) = –3.194, *p* < 0.01] and relative heart weights were significantly decreased [*t*(9.172) = 3.175, *p* < 0.01] in stressed animals compared to non-stressed controls (Supplementary Fig. 1A,B).

**Figure 1:**
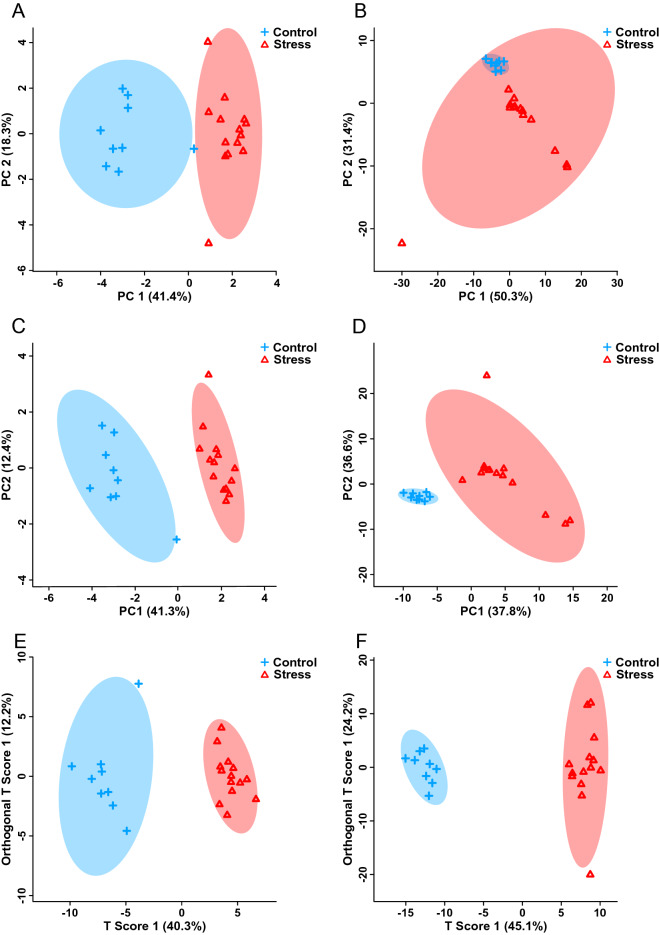
PCA (**A,B**) PLS-DA (**C,D**) and OPLS-DA (**E,F**) scores plots showing statistically significant supervised separation between adult mice exposed to early life stress (n = 14) versus controls (n = 9) for both kidney (**A,C,E**) and heart (**B,D, F**) tissues. Each triangle or cross represents one individual under study, plotted using a list of kidney or heart metabolites found to be statistically significant by a MW test. For the PCA (**A,B**) and PLS-DA (**C,D**) plots, the x- and y-axes show principal components 1 and 2, respectively. For the OPLS-DA plots (**E,F**), the x- and y-axes show the variance explained across and within the groups, respectively. The percentages shown in brackets along each axis indicate the amount of data variance explained by that component.

### Early life stress induced clearly separated metabolic profiles

The kidney and heart analyses included 170 and 347 spectral bins, respectively. A MW test was applied to each comparison group to identify which features led to univariate statistical differences between the groups. These analyses revealed 81 (47.6% of all spectral bins) and 241 (69.5% of all spectral bins) significantly altered features in kidney and heart tissues, respectively. Unsupervised multivariate PCA tests were initially performed using all features. In kidney tissues, separation of the groups was observed in the PCA scores plot, with principal components 1 and 2 accounting for 45.1% and 16.1% of the total variance, respectively (Supplementary Fig. 2). In heart tissues, no separation of groups was observed when considering all bins.

**Figure 2:**
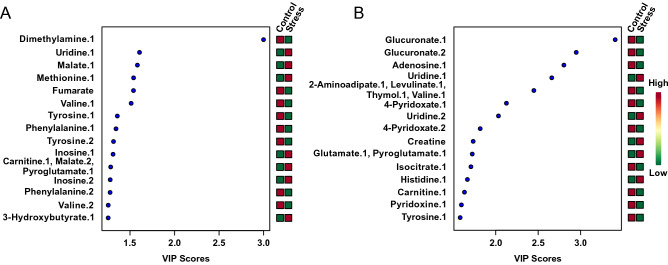
Variable importance in the projection (VIP) plots of adult mice exposed to early life stress (n = 14) and controls (n = 9), showing the relative contribution of metabolites. (**A**) Kidney tissues. (**B**) Heart tissues. High variable importance in the projection values indicate greater contribution of these metabolites to group separation, shown in the PLS-DA plots. Green and red boxes indicate relative metabolite concentration. A score of 1.0 is considered able to discriminate between two phenotypes.

Supervised and unsupervised multivariate statistical tests were then performed using the bins identified as significant from the MW test. In both kidney and heart tissues, the unsupervised PCA scores plots showed clear group separation, with principle component 1 being equal to 41.4% and 50.3% of the total variance and principle component 2 being equal to 18.3% and 31.4% of the total variance, respectively (Fig. 1A,B). The scores plots from supervised PLS-DA (Fig. 1C,D) and OPLS-DA (Fig. 1E,F) tests also both displayed distinct group separation, supporting the PCA findings. Cross-validation and permutation tests validated the observed supervised separation results as a function of early life stress in both kidneys (PLS-DA: *p* < 0.01, R^2^ = 90.2%, Q2 = 83.9%; OPLS-DA: *p* < 0.001, R^2^ = 94.9%, Q2 = 85.9%) and hearts (PLS-DA: *p* < 0.01, R^2^ = 74.4%, Q2 = 56.1%; OPLS-DA: *p* < 0.001, R^2^ = 98.7%, Q2 = 95.5%).

Variable importance in the projection plots (Fig. 2) indicate which variables contributed most to the group separation observed in the PLS-DA scores plot. In kidney tissues, dimethylamine, uridine, and malate contributed most to the separation (Fig. 2A), with variable importance in the projection scores of 3.00, 1.61, and 1.58, respectively (Table 1). In heart tissues, glucuronate, adenosine, and uridine contributed most to the separation (Fig. 2B), with variable importance in the projection scores of 3.41, 2.80, and 2.66, respectively (Table 1). In kidneys, 22/44 (50.0%) unique, significantly altered metabolites (as determined by the MW test) were up-regulated in stressed animals compared to controls, with the remaining metabolites being down-regulated (Supplementary Table 1). In hearts, 54/82 (65.9%) unique, significantly altered metabolites were down-regulated in stressed animals compared to controls, with the remainder being up-regulated (Supplementary Table 1).

**Table 1:** Kidney and heart metabolites found to be most significantly altered by stress in a Mann–Whitney U test (n = 9 control animals; n = 14 stressed animals).

Organ	Metabolite	NMR chemical shift range of bin (ppm)	Mann–Whitney U test	Percent difference	Variable importance in the projection score	Regulation by stress
Kidney	Dimethylamine.1^†^	2.771–2.756	8.25E−05	− 86.21	3.00	Down
	Uridine.1^†^	4.344–4.320	1.82E−02	40.14	1.61	Up
	Malate.1^†^	2.653–2.639	1.38E−04	28.94	1.58	Up
	Methionine.1	2.639–2.630	1.07E−04	27.16	1.54	Up
	Fumarate^†^	6.533–6.519	1.82E−03	− 34.74	1.54	Down
	Valine.1^†^	2.326–2.248	8.25E−05	− 24.00	1.51	Down
	Tyrosine.1^†^	3.051–2.980	1.38E−04	− 20.13	1.36	Down
	Phenylalanine.1	7.813–7.801	1.18E−03	− 24.54	1.34	Down
	Tyrosine.2^†^	2.870–2.854	1.82E−03	− 24.50	1.31	Down
	Inosine.1^†^	4.466–4.441	1.82E−02	− 34.38	1.31	Up
	Carnitine.1^†^, Malate.2^†^, Pyroglutamate.1^†^	2.476–2.429	8.25E−05	18.03	1.28	Up
	Inosine.2^†^	6.817–6.806	2.53E−02	29.34	1.28	Up
	Phenylalanine.2	7.453–7.415	1.18E−03	− 19.81	1.28	Down
	Valine.2^†^	1.058–1.029	1.82E−03	− 19.24	1.26	Down
	3-Hydroxybutyrate.1	0.901–0.886	1.47E−03	22.37	1.25	Up
	Aspartate.1^†^	2.722–2.709	1.38E−04	− 17.36	1.25	Down
	Phenylalanine.3	7.354–7.321	1.18E−03	− 18.58	1.23	Down
	Acetate.1	1.967–1.873	5.97E−04	− 17.93	1.23	Down
	Dimethylamine.2^†^	2.738–2.722	8.25E−05	− 15.33	1.18	Down
	Aspartate.2^†^	2.697–2.686	8.25E−05	− 15.24	1.17	Down
Heart	Glucuronate.1	4.675–4.663	7.51E−04	− 149.75	3.41	Down
	Glucuronate.2	4.663–4.652	7.51E−04	− 143.00	2.95	Down
	Adenosine.1	4.437–4.321	7.51E−04	− 83.06	2.80	Down
	Uridine.1^†^	7.930–7.921	8.25E−05	137.40	2.66	Up
	2-Aminoadipate.1^†^, Levulinate.1, Thymol.1, Valine.1^†^	2.242–2.234	8.25E−05	− 122.46	2.45	Down
	4-Pyridoxate.1^†^	7.938–7.930	8.25E−05	− 131.59	2.13	Down
	Uridine.2^†^	8.079–8.069	8.25E−05	94.58	2.03	Up
	4-Pyridoxate.2^†^	8.114–8.107	8.25E−05	− 109.50	1.82	Down
	Creatine^†^	3.060–3.049	8.25E−05	69.32	1.73	Up
	Glutamate.1^†^, Pyroglutamate.1^†^	2.415–2.402	8.25E−05	71.47	1.72	Up
	Isocitrate.1	2.564–2.551	8.25E−05	− 68.36	1.71	Down
	Histidine.1^†^	7.165–7.145	7.51E−04	83.75	1.67	Up
	Carnitine.1^†^	2.476–2.464	8.25E−05	− 64.95	1.63	Down
	Pyridoxine.1	2.485–2.476	8.25E−05	− 62.29	1.60	Down
	Tyrosine.1^†^	7.180–7.165	8.25E−05	− 86.67	1.58	Down
	N-Methylhydantoin.1, N,N-Dimethylglycine.1, Trimethylamine.1	2.907–2.897	8.25E−05	− 59.07	1.53	Down
	Carnitine.2^†^, 4-Pyridoxate.3^†^	2.464–2.453	8.25E−05	− 56.71	1.51	Down
	Glutamate.2^†^, Pyroglutamate.2^†^, Succinate.1^†^	2.402–2.390	8.25E−05	55.08	1.50	Up
	Levulinate.2	2.789–2.771	8.25E−05	− 57.44	1.48	Down
	Levulinate.3	2.771–2.754	8.25E−05	− 54.01	1.47	Down

**Figure 3:**
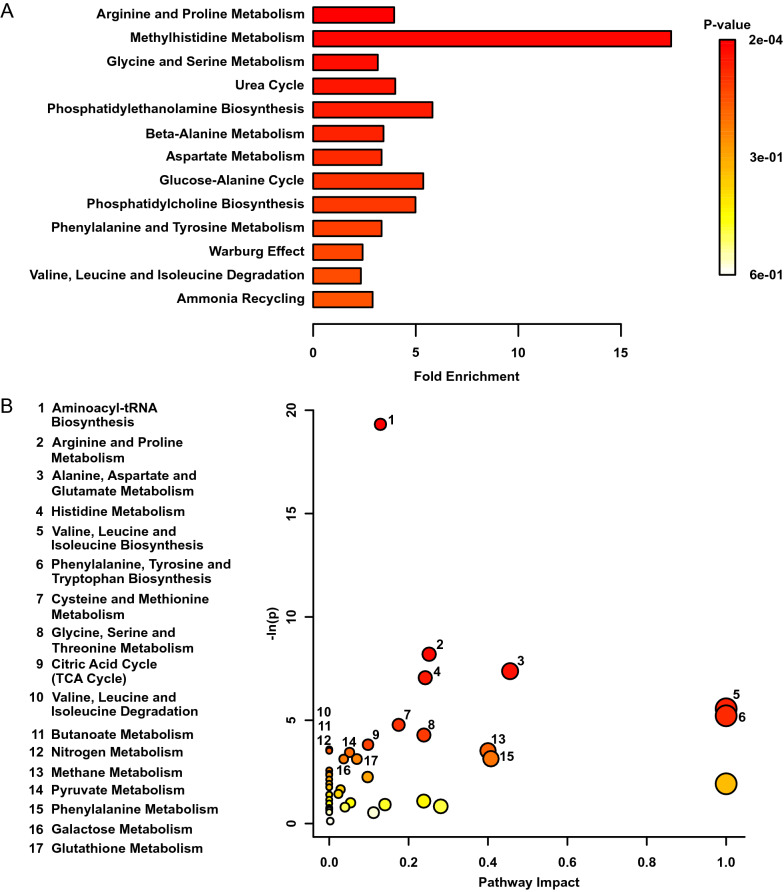
(**A**) Metabolite Set Enrichment Analysis plot of adult mice exposed to early life stress (n = 14) compared to controls (n = 9). (**B**) Metabolomic Pathway Analysis showing all matched pathways according to* p*-values from pathway enrichment analysis and pathway impact values in kidney tissues. A higher value on the y-axis indicates a lower* p*-value. The x-axis gives the Pathway Impact. Only metabolic pathways with *p* < 0.05 are labeled. This figure was created using the lists of metabolites identified as significant in a MW test.

Top 20 variable importance in the projection scores, shown in descending order, correspond to Fig. 3A,B. Metabolite regulation is shown as a function of relative concentration in high-EPS individuals. Metabolites for which more than one NMR resonance peak was identified as significant are represented as metabolite.1, metabolite.2, … metabolite.n.

^†^Indicates metabolites that were differentially regulated in both kidney and heart tissues.

### Early life stress permanently altered metabolic pathway function

**Figure 4:**
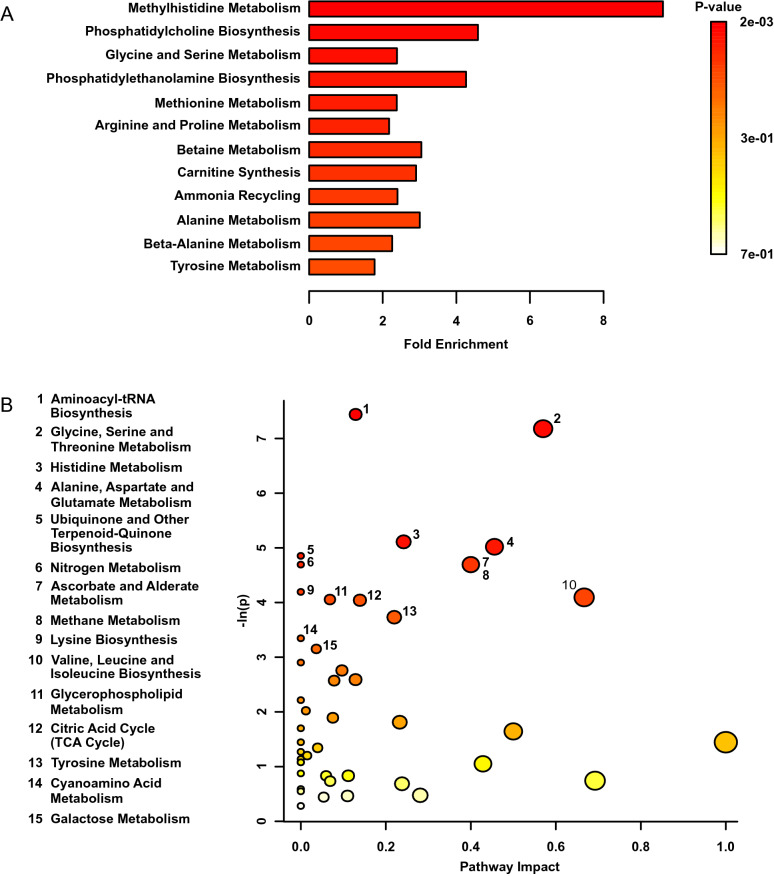
(**A**) Metabolite Set Enrichment Analysis plot of adult mice exposed to early life stress (n = 14) compared to controls (n = 9). (**B**) Metabolomic Pathway Analysis showing all matched pathways according to* p*-values from pathway enrichment analysis and pathway impact values in heart tissues. A higher value on the y-axis indicates a lower* p*-value. The x-axis gives the Pathway Impact. Only metabolic pathways with *p* < 0.05 are labeled. This figure was created using the lists of metabolites identified as significant in a MW test.

A genome-wide network model of mouse metabolism was used to investigate metabolite sets altered as a result of early life stress (Figs. 3 and 4). In kidney tissues, early postnatal stress exposure most significantly affected arginine and proline metabolism (*p* < 0.001), methylhistidine metabolism (*p* < 0.001), as well as glycine and serine metabolism (*p* < 0.01) (Fig. 3A). Additionally, numerous energy metabolism systems were altered, including pathways in aminoacyl-tRNA biosynthesis (*p* < 0.00000001), arginine and proline metabolism (*p* < 0.001), alanine, aspartate, and glutamate metabolism (*p* < 0.001), and valine, leucine, and isoleucine degradation (*p* < 0.05) (Fig. 3B). In heart tissues, early life stress most significantly altered methylhistidine metabolism (*p* < 0.01), phosphatidylcholine biosynthesis (*p* < 0.01), as well as glycine and serine metabolism (*p* < 0.01) (Fig. 4A). Pathway topology analyses (Fig. 4B) revealed significant effects on aminoacyl-tRNA biosynthesis (*p* < 0.001), glycine, serine, and threonine metabolism (*p* < 0.001), histidine metabolism (*p* < 0.01), and valine, leucine, and isoleucine biosynthesis (*p* < 0.05).

### Early life stress-induced behavioural phenotype was associated with metabolic function

**Figure 5:**
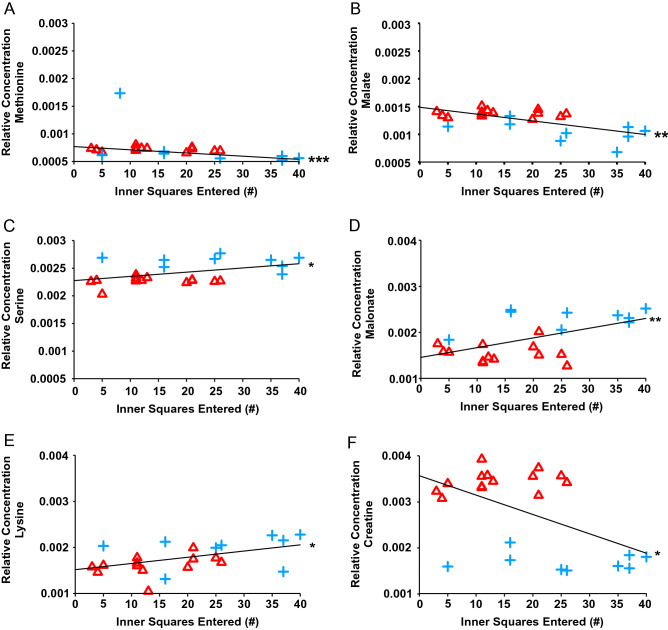
Pearson correlations to assess the relationship between anxiety-like behaviour and the relative concentrations of (**A**) methionine, (**B**) malate, (**C**) serine, (**D**) malonate, (**E**) lysine, and (**F**) creatine in either kidney (**A,B,C**) or heart (**D,E,F**) tissues (Blue Cross, n = 9 control animals; Red Triangle, n = 14 stressed animals). There were negative correlations between the number of center squares entered and methionine (*r* = –0.66, *p* = 0.0007), malate (*r* = –0.64, *p* = 0.0011), and creatine (*r* = –0.52, *p* = 0.0114), indicating that a higher anxiety-like state was linked to higher methionine, malate, and creatine concentrations. There were positive correlations between the number of center squares entered and serine (*r* = 0.45, *p* = 0.0293), malonate (*r* = 0.57, *p* = 0.0050), and lysine (*r* = 0.49, *p* = 0.0173), indicating that a higher anxiety-like state was linked to lower serine, malonate, and lysine concentrations.

Open field locomotor profiles revealed increased anxiety-like behaviour in stressed animals compared to non-stressed controls, as reflected by the number of squares traversed in the centre of the open field arena [*t*(21) = 3.069, *p* = 0.0060]. Sixteen and 117 features correlated significantly with increased anxiety-like behaviours in kidney and heart tissues, respectively (Supplementary Table 2). Of interest, the relationship between the number of central squares traversed and the relative concentrations of methionine, malate, and serine indicated a positive correlation for serine (*r* = 0.45, *p* = 0.029) and negative correlations for methionine (*r* = –0.66, *p* = 0.00066) and malate (*r* = –0.64, *p* = 0.0011) in kidney tissues (Fig. 5A, Table 2). The relationship between the number of central squares traversed and the relative concentrations of malonate, lysine, and creatine indicated a negative correlation for creatine (*r* = –0.52, *p* = 0.011) and positive correlations for malonate (*r* = 0.57, *p* = 0.0050) and lysine (*r* = 0.49, *p* = 0.017) in heart tissues (Fig. 5B, Table 2).

**Table 2:** Kidney and heart metabolites found to be most significantly correlated to anxiety-like behaviour.

Organ	Metabolite	*r*	*p*	Correlation
Kidney	Methionine.1	− 0.66	0.0007	Negative
	Malate.1	− 0.64	0.0011	Negative
	Malate.3	− 0.61	0.0019	Negative
	Glucose.1	0.60	0.0024	Positive
	2-Aminoadipate^‡^	0.57	0.0042	Positive
	Cystine	0.56	0.0057	Positive
	Dimethylamine.1	0.54	0.0076	Positive
	Glutamate.1^‡^, Methionine.2	− 0.53	0.0097	Negative
	3-Hydroxybutyrate.1	− 0.50	0.0150	Negative
	Glucose.3	0.49	0.0187	Positive
	Serine.1	0.45	0.0293	Positive
	Choline.5^‡^	− 0.45	0.0307	Negative
	Choline.1^‡^	− 0.44	0.0373	Negative
	Alanine^‡^	0.43	0.0425	Positive
	Glucose.4	0.43	0.0397	Positive
Heart	2-Hydroxybutyrate.1	0.65	0.0009	Positive
	Alanine.3^‡^, N-Phenylacetylglycine.2, O-Acetylcholine.1	− 0.63	0.0012	Negative
	3-Hydroxyisovalerate.2	0.63	0.0014	Positive
	Cholate.1	0.61	0.0019	Positive
	Formate	0.61	0.0018	Positive
	Galactitol.1	0.61	0.0020	Positive
	Methylmalonate.2	0.61	0.0022	Positive
	Thymol.7	0.61	0.0018	Positive
	4-Pyridoxate.1	0.60	0.0025	Positive
	Cholate.3	0.60	0.0025	Positive
	3-Methylglutarate.1	0.60	0.0022	Positive
	Methylmalonate.1	0.59	0.0033	Positive
	Alanine.4^‡^, N-Phenylacetylglycine.4, O-Acetylcholine.2	− 0.59	0.0033	Negative
	Glutamate.8^‡^	− 0.59	0.0033	Negative
	2-Aminoadipate.1^‡^, Levulinate.1, Thymol.1, Valine.1	0.58	0.0038	Positive
	Malonate.1	0.57	0.0050	Positive
	Creatine	− 0.52	0.0114	Negative
	Lysine.1	0.49	0.0173	Positive

Pearson correlations were used to assess the relationship between behaviours indicative of heightened anxiety (i.e., more central squares entered in the open field) and relative concentrations of metabolites found to be significantly altered by stress in a Mann–Whitney U test (n = 9 control animals; n = 14 stressed animals). Top 15 *r* values, shown in descending order, correspond to Fig. 5. In hearts, the bottom three entries correspond to additional metabolites shown to have an association with adverse mental health outcomes. Positive correlations indicate that a higher anxiety-like state was linked to lower metabolite concentrations, while negative correlations indicate that a higher anxiety-like state was linked to higher metabolite concentrations. Metabolites for which more than one NMR resonance peak was identified are represented as metabolite.1, metabolite.2, … metabolite.n.

^‡^Indicates metabolites that were significantly correlated to anxious behaviour in both kidney and heart tissues.

## Discussion

The perinatal environment is a critical determinant of lifelong NCD risk. Here, we found that multidimensional early life stress was linked to chronic cardiorenal metabolic pathologies in adulthood based on stressed animals having altered (i) organ weights, (ii) affective state, and (iii) metabolites and/or metabolic pathways linked to adverse mental health outcomes and metabolic illness, such as cardiorenal syndrome, insulin resistance, diabetes, and obesity. Experience-dependent metabolic markers in heart and kidney are involved in energy metabolism, branched-chain amino acid biosynthesis and degradation, methylhistidine metabolism, arginine and proline metabolism, and glycine and serine metabolism. Moreover, many of the metabolites found to be significantly altered by stress belong to metabolic pathways involved in aminoacyl-tRNA biosynthesis, supporting a link between early environmental insults and NCD risk later in life.

Previous studies have linked perinatal stress to abnormal HPA axis function in the offspring, along with neuronal and receptor loss in brain areas associated with the control of stress response and affective state^34^. Interestingly, these hormonal changes are associated with epigenetic changes, including altered microRNA expression, along with HPA axis reprogramming^35^. The present findings support these earlier studies by linking early life stress to cardiorenal remodeling. The observed changes in heart and kidney weights suggest a permanent reprogramming of physiological, metabolic, and epigenetic processes manifesting as downstream alterations in cardiorenal phenotype and function. Indeed, the development of renal pathology was associated with increased kidney weight. Absolute kidney weight in animals and humans is attributable to endothelial cell proliferation and intrarenal lipid deposition that impair intrarenal hemodynamics^36,37^. Stress-induced kidney hypertrophy is also correlated with higher blood pressure and subsequent hypertensive renal damage^38^. Similarly, cardiac remodeling and hypertrophy are pathologically associated with NCDs such as diabetes, obesity, and CVDs^39^. By contrast, relative heart weight is decreased in diabetes due to myocyte loss and inadequate reactive hypertrophy of the remaining cells^40^, which is also related to deteriorated heart contractile function^41^ and impaired insulin signaling^42^. Thus, the present data suggest an association between early life stress and cardiac atrophy and risk of metabolic disorders such as diabetes.

Early life stress significantly changed 81 metabolites in kidney tissues and 241 metabolites in heart tissues. In both heart and kidney, stress most drastically altered pathways involved in aminoacyl-tRNA biosynthesis. Thus, while alterations in individual metabolites were not identical across tissue types, aggregate stress-induced metabolic changes ultimately led to a shared cardiorenal phenotype. Accordingly, previous work demonstrated mitochondrial dysfunction, cardiorenal pathologies, and impaired aminoacyl-tRNA biosynthesis in response to environmental insults^26,39,43,44^.

Several metabolites that obtained a significant variable importance in the projection score in both heart and kidney tissues are involved in global hemodynamics. Abnormal levels of metabolites may mediate vasoconstriction and vasodilation, such as uridine and adenosine, and impair hemodynamics and hypertension and are risk factors for non-communicable cardiovascular and kidney diseases. Uridine, which has vasoconstrictive actions, was up-regulated in both heart and kidney, while adenosine, an inhibitor of vasoconstriction, was down-regulated. Indeed, hypertension is characterized by a sustained increase in total peripheral vascular resistance, suggestive of a primary cause in hypertension^45^. In kidneys, dimethylamine, a product of the hydrolysis of asymmetric dimethylarginine^46^, contributed to group separations. Kidneys are a major extraction site for asymmetric dimethylarginine^47^ and renal dysfunction promotes accumulation of asymmetric dimethylarginine and reduced dimethylamine^46^.

Multiple stress-responsive metabolites are involved in energy metabolism. Creatine, a natural regulator of energy homeostasis, was up-regulated by stress and correlated strongly with increased anxiety-like behaviours. The Krebs cycle, taking place in the mitochondrial matrix, acts as a nexus for the integration of several catabolic and anabolic pathways. Abnormal levels of metabolites involved in the citric acid cycle, such as malate and malonate, can lead to mitochondrial dysfunction. Stress-induced divergent mitochondrial pathways have been implicated in the regulation of integrated central nervous system function, with aberrant energy metabolism playing a key role in the pathophysiological underpinnings of anxiety disorders. Oxidative stress, involved in the pathogenesis of mental health disorders^48^, cardiovascular and kidney diseases^49^, is caused by altered mitochondrial energy pathways leading to overabundance of oxidative stress compounds. In both animals and humans, stress-induced anxiety alters levels of Krebs cycle intermediates, which subsequently exacerbates oxidative damage.

In heart tissues, glucuronate, a product of the oxidative cleavage of myo-inositol catalyzed by myo-inositol oxygenase, was highly significant in contributing to unsupervised and supervised separations. Both glucuronate and myo-inositol are involved in inositol metabolism, supporting the involvement of this biochemical pathway in the mediation of stress-induced metabolic changes. Abnormalities in inositol metabolism have been implicated in insulin resistance and long-term microvascular complications in diabetes^50^. In renal tissues, myo-inositol depletion has been associated with diabetic nephropathy through the activation of fibronectin^50^. Myo-inositol has also been found to decrease in the prefrontal cortex and cerebrospinal fluid in affective disorders^51,52^. Interestingly, glucuronate is markedly reduced in the urine of perinatally-stressed rats with atherosclerosis, suggesting a link to atherogenesis following early life stress^43^.

The open field observations revealed an anxiogenic effect of early life stress. Increased anxiety-like behaviours in stressed animals correlated with the relative concentrations of serine and methionine in their kidneys. Stress down-regulated serine, a co-agonist of ionotropic N-methyl-D-aspartic acid receptor activation and involved in affective behaviour, with serine-depleted mice exhibiting more anxious behaviour and impaired cognitive function^53^. Conversely, elevated brain serine is linked to anxiety-like behaviours, with chronic dietary serine supplementation also having anxiolytic consequences^54^. Interestingly, plasma serine levels correlate with glomerular filtration ratio^55^. The renal reabsorption of serine is sensitive to the presence of chronic kidney disease, with the combination of plasma serine and urinary dynamics effectively distinguishing chronic kidney disease from non-diseased^55^.

Stress also up-regulated methionine, a sulfur-containing donor of methyl groups. Elevated methionine levels have been implicated in the pathogenesis of disorders linked to oxidative stress. In mice, a high-methionine diet is associated with oxidative stress in cardiac tissues due to increased levels of lectin-like oxidized low-density lipoprotein receptor-1 (LOX-1) and superoxide dismutase 1 (SOD1)^44^. Abnormally high levels of methionine also have anxiogenic effects mediated by oxidative stress^56^. Thus, stress-induced methionine increases may provide a mechanism underlying the comorbidity of cardiorenal and affective disorders.

The increased prevalence of anxiety-like behaviours in stressed animals correlated with low concentrations of lysine in heart tissues. In both animals and humans, prolonged dietary lysine inadequacy can elevate stress-induced anxiety while lysine fortification can reduce chronic anxiety^57^. In a rural Syrian population dietary lysine supplementation was found to lower blood cortisol levels and sympathetic arousal in response to stress^57^. Anxiogenic consequences of lysine deficiency may be mediated by serotonin alterations in the central amygdala^58^, with lysine acting as a partial serotonin receptor 4 antagonist. Since abnormal concentrations of lysine have been associated with diabetes^59^, abnormal altered lysine metabolism may also be a factor in chronic disease etiology.

Metabolite set enrichment analysis was used to identify patterns of metabolite concentration changes in a biologically meaningful framework^60^. The most significant pathway altered in heart tissues was methylhistidine metabolism, with 3 metabolite hits, whereas in kidney tissues, this was the case for arginine and proline metabolism, with 9 metabolite hits. Significant pathways in the kidney metabolite set enrichment analysis, apart from arginine and proline metabolism, included methylhistidine metabolism as well as valine, leucine, and isoleucine degradation. In the heart metabolite set enrichment analysis, significant pathways included glycine and serine metabolism, methionine metabolism, and arginine and proline metabolism. 3-Methylhistidine, a constituent of actin and myosin released via protein degradation, was up-regulated in response to stress. Methylhistidine metabolism serves as an indicator of muscle myofibrillar protein breakdown, with an increase in 3-methylhistidine release in muscle atrophy^61^. Disruptions in methionine, glycine and serine metabolisms support the finding that stressed animals are at a higher risk for developing cardiorenal and affective disorders. Furthermore, decreased arginine availability due to perturbed renal biosynthesis is causally implicated in nitric oxide deficiency, which contributes to cardiovascular events and kidney damage^62^.

Consistent with metabolite set enrichment analysis results and variable importance in the projection scores, both heart and kidney pathway topology analyses revealed changes in valine, leucine, and isoleucine biosynthesis and degradation. This draws attention to a possible metabolic dysregulation of branched-chain amino acids in stressed animals. Abnormal branched-chain amino acid metabolism has been implicated in the pathogenesis of insulin resistance, obesity, and type 2 diabetes mellitus. Altered concentrations of branched-chain amino acids promotes oxidative stress and inflammation of peripheral blood mononuclear cells via mTORC1 activation^63,64^, thus contributing to the pro-inflammatory and oxidative status observed in many pathophysiological conditions.

Stress also altered the aminoacyl-tRNA biosynthesis pathway, which is central for biosynthesis and kinetics of mRNA translation. Accordingly, changes in aminoacyl-tRNA biosynthesis have been causally implicated in the pathogenesis of mitochondrial metabolic and congenital heart diseases. In diabetes, both hyperglycemia and hyperinsulinemia stimulate the accumulation of mutations in mitochondrial tRNAs, the substrates of aminoacyl-tRNA synthetases, by inducing oxidative stress^65^. Altogether, the present results suggest that multidimensional early life stress increases the risk for developing diabetes later in life.

The present findings provide novel insights into the mechanisms underlying early life stress-induced disease vulnerability by linking a cardiorenal metabolic stress phenotype to chronic heart and kidney dysfunction and impaired mental health. These data also suggest a role for transportation stress in the etiology of long-term health complications in laboratory animals and livestock^29–31^. The metabolic changes arguably reflect underlying epigenetic modifications and associated cellular functions that manifest as downstream alterations to the metabolome. Clearly recognizable metabolic biomarkers of disease open new personalized medicine strategies for early detection and diagnosis of chronic NCDs in clinical populations.

## Supplementary information

Supplementary Information.

Supplementary Figure 1.

Supplementary Figure 2.

Supplementary Tables.
