# Supplementary material for: Cardiorenal metabolic biomarkers link early life stress to risk of non-communicable diseases and adverse mental health outcomes
Source: Sci Rep. 2020 Aug 6;10:13295. doi: 10.1038/s41598-020-69866-3 (PMC7413400; doi:10.1038/s41598-020-69866-3)
Supplement: Supplementary file 1 — Supplementary Information. [file 41598_2020_69866_MOESM1_ESM.docx]

**Cardiorenal Metabolic Biomarkers Link Early-Life Stress to Risk of Non-Communicable Diseases and Adverse Mental Health Outcomes**

*Janet Poplawski^1^, Ana Radmilovic^1^, Tony D. Montina^2*^, Gerlinde A.S. Metz^1*^*

^1^ Canadian Centre for Behavioural Neuroscience, Department of Neuroscience, University of Lethbridge, 4401 University Drive, Lethbridge, AB T1K 3M4, Canada

^2^ Department of Chemistry and Biochemistry, University of Lethbridge, 4401 University Drive, Lethbridge, AB T1K 3M4, Canada

**Running Title:** Cardiorenal Metabolomic Biomarkers

**Category:** Primary Research Paper
