# Supplementary figures and images for: Cardiorenal metabolic biomarkers link early life stress to risk of non-communicable diseases and adverse mental health outcomes
Source: Sci Rep. 2020 Aug 6;10:13295. doi: 10.1038/s41598-020-69866-3 (PMC7413400; doi:10.1038/s41598-020-69866-3)

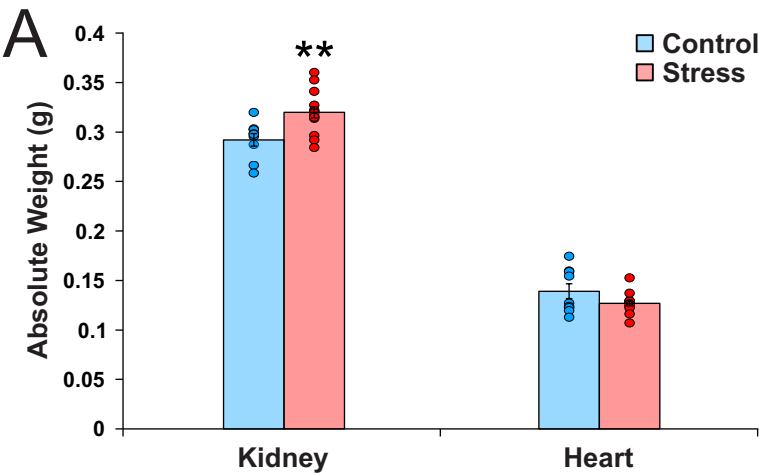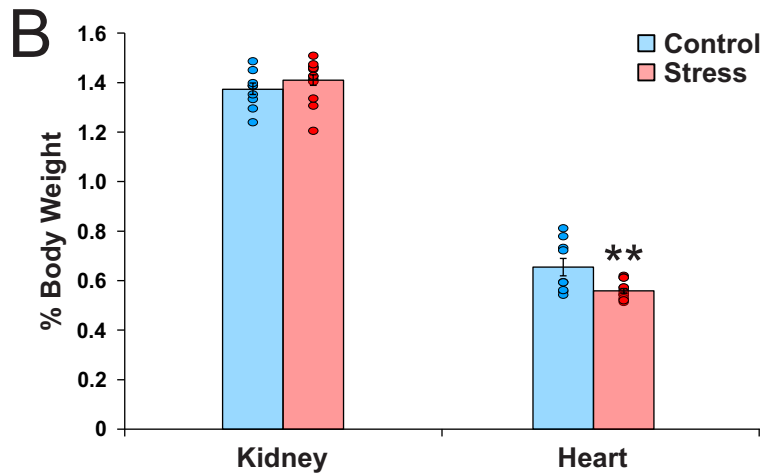

Supplement: Supplementary file 2 — Supplementary Figure 1. [file 41598_2020_69866_MOESM2_ESM.pdf]

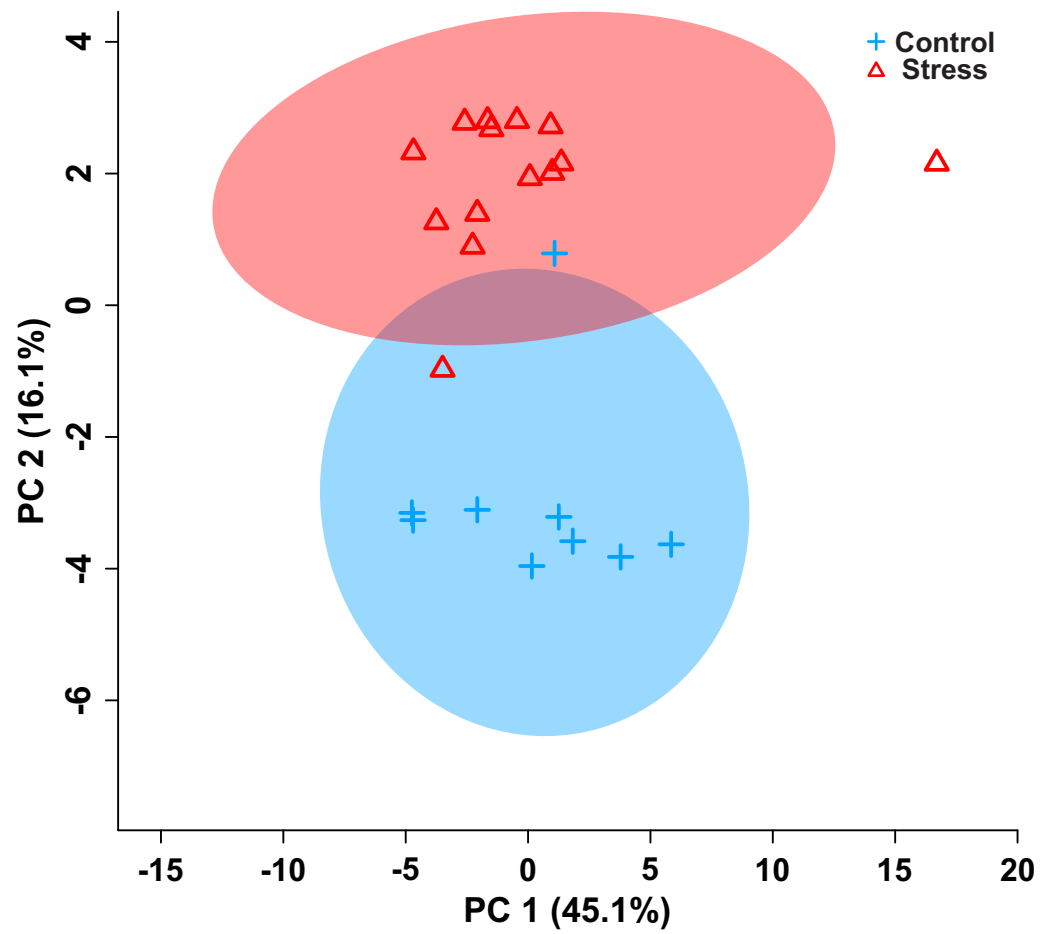

Supplement: Supplementary file 3 — Supplementary Figure 2. [file 41598_2020_69866_MOESM3_ESM.pdf]
