# Supplementary material for: Cardiorenal metabolic biomarkers link early life stress to risk of non-communicable diseases and adverse mental health outcomes
Source: Sci Rep. 2020 Aug 6;10:13295. doi: 10.1038/s41598-020-69866-3 (PMC7413400; doi:10.1038/s41598-020-69866-3)
Supplement: Supplementary file 4 — Supplementary Tables. [file 41598_2020_69866_MOESM4_ESM.docx]

**Supplementary Figures/Tables**

| **Organ** | **Metabolite** | **NMR Chemical Shift Range of Bin (ppm)** | **Mann-Whitney U Test** | **Percent Difference** | **Variable Importance in the Projection Score** | **Regulation by Stress** |
| --- | --- | --- | --- | --- | --- | --- |
| Kidney | Dimethylamine.1^†^ | 2.771–2.756 | 8.25E-05 | –86.21 | 3.00 | Down |
|  | Uridine.1^†^ | 4.344–4.320 | 1.82E-02 | 40.14 | 1.61 | Up |
|  | Malate.1^†^ | 2.653–2.639 | 1.38E-04 | 28.94 | 1.58 | Up |
|  | Methionine.1 | 2.639–2.630 | 1.07E-04 | 27.16 | 1.54 | Up |
|  | Fumarate^†^ | 6.533–6.519 | 1.82E-03 | –34.74 | 1.54 | Down |
|  | Valine.1^†^ | 2.326–2.248 | 8.25E-05 | –24.00 | 1.51 | Down |
|  | Tyrosine.1^†^ | 3.051–2.980 | 1.38E-04 | –20.13 | 1.36 | Down |
|  | Phenylalanine.1 | 7.813–7.801 | 1.18E-03 | –24.54 | 1.34 | Down |
|  | Tyrosine.2^†^ | 2.870–2.854 | 1.82E-03 | –24.50 | 1.31 | Down |
|  | Inosine.1^†^ | 4.466–4.441 | 1.82E-02 | –34.38 | 1.31 | Up |
|  | Carnitine.1^†^, Malate.2^†^, Pyroglutamate.1^†^ | 2.476–2.429 | 8.25E-05 | 18.03 | 1.28 | Up |
|  | Inosine.2^†^ | 6.817–6.806 | 2.53E-02 | 29.34 | 1.28 | Up |
|  | Phenylalanine.2 | 7.453–7.415 | 1.18E-03 | –19.81 | 1.28 | Down |
|  | Valine.2^†^ | 1.058–1.029 | 1.82E-03 | –19.24 | 1.26 | Down |
|  | 3-Hydroxybutyrate.1 | 0.901–0.886 | 1.47E-03 | 22.37 | 1.25 | Up |
|  | Aspartate.1^†^ | 2.722–2.709 | 1.38E-04 | –17.36 | 1.25 | Down |
|  | Phenylalanine.3 | 7.354–7.321 | 1.18E-03 | –18.58 | 1.23 | Down |
|  | Acetate.1 | 1.967–1.873 | 5.97E-04 | –17.93 | 1.23 | Down |
|  | Dimethylamine.2^†^ | 2.738–2.722 | 8.25E-05 | –15.33 | 1.18 | Down |
|  | Aspartate.2^†^ | 2.697–2.686 | 8.25E-05 | –15.24 | 1.17 | Down |
| Heart | Glucuronate.1 | 4.675–4.663 | 7.51E-04 | –149.75 | 3.41 | Down |
|  | Glucuronate.2 | 4.663–4.652 | 7.51E-04 | –143.00 | 2.95 | Down |
|  | Adenosine.1 | 4.437–4.321 | 7.51E-04 | –83.06 | 2.80 | Down |
|  | Uridine.1^†^ | 7.930–7.921 | 8.25E-05 | 137.40 | 2.66 | Up |
|  | 2-Aminoadipate.1^†^, Levulinate.1, Thymol.1, Valine.1^†^ | 2.242–2.234 | 8.25E-05 | –122.46 | 2.45 | Down |
|  | 4-Pyridoxate.1^†^ | 7.938–7.930 | 8.25E-05 | –131.59 | 2.13 | Down |
|  | Uridine.2^†^ | 8.079–8.069 | 8.25E-05 | 94.58 | 2.03 | Up |
|  | 4-Pyridoxate.2^†^ | 8.114–8.107 | 8.25E-05 | –109.50 | 1.82 | Down |
|  | Creatine^†^ | 3.060–3.049 | 8.25E-05 | 69.32 | 1.73 | Up |
|  | Glutamate.1^†^, Pyroglutamate.1^†^ | 2.415–2.402 | 8.25E-05 | 71.47 | 1.72 | Up |
|  | Isocitrate.1 | 2.564–2.551 | 8.25E-05 | –68.36 | 1.71 | Down |
|  | Histidine.1^†^ | 7.165–7.145 | 7.51E-04 | 83.75 | 1.67 | Up |
|  | Carnitine.1^†^ | 2.476–2.464 | 8.25E-05 | –64.95 | 1.63 | Down |
|  | Pyridoxine.1 | 2.485–2.476 | 8.25E-05 | –62.29 | 1.60 | Down |
|  | Tyrosine.1^†^ | 7.180–7.165 | 8.25E-05 | –86.67 | 1.58 | Down |
|  | N-Methylhydantoin.1, N,N-Dimethylglycine.1, Trimethylamine.1 | 2.907–2.897 | 8.25E-05 | –59.07 | 1.53 | Down |
|  | Carnitine.2^†^, 4-Pyridoxate.3^†^ | 2.464–2.453 | 8.25E-05 | –56.71 | 1.51 | Down |
|  | Glutamate.2^†^, Pyroglutamate.2^†^, Succinate.1^†^ | 2.402–2.390 | 8.25E-05 | 55.08 | 1.50 | Up |
|  | Levulinate.2 | 2.789–2.771 | 8.25E-05 | –57.44 | 1.48 | Down |
|  | Levulinate.3 | 2.771–2.754 | 8.25E-05 | –54.01 | 1.47 | Down |

**Supplementary Table 1.** Kidney and heart metabolites found to be most significantly altered by stress in a Mann-Whitney U test (n=9 control animals; n=14 stressed animals). Top 20 variable importance in the projection scores, shown in descending order, correspond to Fig. 3A, B. Metabolite regulation is shown as a function of relative concentration in high-EPS individuals. Metabolites for which more than one NMR resonance peak was identified as significant are represented as metabolite.1, metabolite.2, … metabolite.n. ^†^Indicates metabolites that were differentially regulated in both kidney and heart tissues.

| **Organ** | **Metabolite** | ***r*** | ***p*** | **Correlation** |
| --- | --- | --- | --- | --- |
|  | Methionine.1 | –0.66 | 0.0007 | Negative |
|  | Malate.1 | –0.64 | 0.0011 | Negative |
|  | Malate.3 | –0.61 | 0.0019 | Negative |
|  | Glucose.1 | 0.60 | 0.0024 | Positive |
|  | 2-Aminoadipate^‡^ | 0.57 | 0.0042 | Positive |
|  | Cystine | 0.56 | 0.0057 | Positive |
|  | Dimethylamine.1 | 0.54 | 0.0076 | Positive |
| Kidney | Glutamate.1^‡^, Methionine.2 | –0.53 | 0.0097 | Negative |
|  | 3-Hydroxybutyrate.1 | –0.50 | 0.0150 | Negative |
|  | Glucose.3 | 0.49 | 0.0187 | Positive |
|  | Serine.1 | 0.45 | 0.0293 | Positive |
|  | Choline.5^‡^ | –0.45 | 0.0307 | Negative |
|  | Choline.1^‡^ | –0.44 | 0.0373 | Negative |
|  | Alanine^‡^ | 0.43 | 0.0425 | Positive |
|  | Glucose.4 | 0.43 | 0.0397 | Positive |
|  | 2-Hydroxybutyrate.1 | 0.65 | 0.0009 | Positive |
|  | Alanine.3^‡^, N-Phenylacetylglycine.2, O-Acetylcholine.1 | –0.63 | 0.0012 | Negative |
|  | 3-Hydroxyisovalerate.2 | 0.63 | 0.0014 | Positive |
|  | Cholate.1 | 0.61 | 0.0019 | Positive |
|  | Formate | 0.61 | 0.0018 | Positive |
|  | Galactitol.1 | 0.61 | 0.0020 | Positive |
|  | Methylmalonate.2 | 0.61 | 0.0022 | Positive |
|  | Thymol.7 | 0.61 | 0.0018 | Positive |
|  | 4-Pyridoxate.1 | 0.60 | 0.0025 | Positive |
| Heart | Cholate.3 | 0.60 | 0.0025 | Positive |
|  | 3-Methylglutarate.1 | 0.60 | 0.0022 | Positive |
|  | Methylmalonate.1 | 0.59 | 0.0033 | Positive |
|  | Alanine.4^‡^, N-Phenylacetylglycine.4, O-Acetylcholine.2 | –0.59 | 0.0033 | Negative |
|  | Glutamate.8^‡^ | –0.59 | 0.0033 | Negative |
|  | 2-Aminoadipate.1^‡^, Levulinate.1, Thymol.1, Valine.1 | 0.58 | 0.0038 | Positive |
|  | Malonate.1 | 0.57 | 0.0050 | Positive |
|  | Creatine | –0.52 | 0.0114 | Negative |
|  | Lysine.1 | 0.49 | 0.0173 | Positive |

**Supplementary Table 2.** Kidney and heart metabolites found to be most significantly correlated to anxiety-like behaviour. Pearson correlations were used to assess the relationship between behaviours indicative of heightened anxiety (i.e., more central squares entered in the open field) and relative concentrations of metabolites found to be significantly altered by stress in a Mann-Whitney U test (n=9 control animals; n=14 stressed animals). Top 15 *r* values, shown in descending order, correspond to Fig. 5. In hearts, the bottom three entries correspond to additional metabolites shown to have an association with adverse mental health outcomes. Positive correlations indicate that a higher anxiety-like state was linked to lower metabolite concentrations, while negative correlations indicate that a higher anxiety-like state was linked to higher metabolite concentrations. Metabolites for which more than one NMR resonance peak was identified are represented as metabolite.1, metabolite.2, … metabolite.n. ^‡^Indicates metabolites that were significantly correlated to anxious behaviour in both kidney and heart tissues.

**Figure Captions**

**Supplementary Figure 1.** Early life stress alters heart and kidney weights in adulthood, as indicated by an independent samples *t*-test. (**A**) Absolute organ weights. (**B**) Organ weights as a percentage of total body weight. Animals exposed to stress (n=14) showed significantly increased absolute kidney weights and significantly decreased relative heart weights at P50 compared to non-stressed controls (n=9). *Asterisks* indicate significances: ***p*<0.01. *Error bars* represent ± SEM.

**Supplementary Figure 2.** PCA scores plot showing statistically significant unsupervised separation between adult mice exposed to early life stress (n=14) and controls (n=9) for kidney tissues. Each triangle or cross represents one individual under study, plotted using all kidney metabolites. The x- and y-axes show principal components 1 and 2, respectively. The percentages shown in brackets along each axis indicate the amount of data variance explained by that component.
